# Psychiatric features of two cases of anti-NMDAR autoimmune encephalitis

**DOI:** 10.47626/1516-4446-2023-3430

**Published:** 2024-03-01

**Authors:** Bárbara Ferreira Althoff, Anne Orgler Sordi, Lucas Primo de Carvalho Alves, Lisieux Elaine de Borba Telles

**Affiliations:** 1 Hospital de Clínicas de Porto Alegre (HCPA), Porto Alegre, RS, Brazil; 2 Departamento de Psiquiatria, HCPA, Porto Alegre, RS, Brazil; 3 Hospital Materno Infantil Presidente Vargas, Porto Alegre, RS, Brazil; 4 Universidade Federal do Rio Grande do Sul, Porto Alegre, RS, Brazil

Autoimmune anti-NMDAR encephalitis is a serious, underrecognized, rapidly progressive, and potentially fatal yet treatable condition whose diagnostic criteria were defined in 2016.^1^,^2^ Up to 90% of cases begin with psychiatric symptoms, and 77% are initially evaluated by psychiatrists, making it challenging to distinguish from a primary psychiatric disorder.^2^,^3^ This correspondence aims to emphasize anti-NMDAR encephalitis as a differential diagnosis in patients with subacute psychiatric manifestations.

We present two clinical cases treated at university general hospitals in Rio Grande do Sul, Brazil, followed by an integrative literature review in the PubMed and SciELO databases, conducted in 2020 and 2023. In both cases, the patient or legal guardian provided written informed consent.

The first case, a 25 year-old woman, was admitted to a psychiatric unit due to mutism and catatonia; the second, an 11 year-old boy, was admitted to a pediatrics unit for suicide risk and aggression towards others. Over a period of 4 or more weeks, they both developed neuropsychiatric alterations after initial depressive symptomatology linked to recent family losses. Their neuropsychiatric presentation included disorganized behavior with recurrent agitation, hypersexualization, psychosis, unprovoked laughter, aggression towards others, as well as cognitive changes, orofacial movement disorders, speech disturbances, impaired writing, and progressive decline in self-care and autonomy. In terms of general clinical symptoms, the female patient experienced tonic-clonic epileptic seizures and dysautonomia, while the male patient presented with flu-like symptoms at onset. Neither had a history of psychiatric disorders, and typical findings were absent in complementary exams (i.e., electroencephalogram, blood work, and magnetic resonance imaging were not suggestive) (Box 1). The diagnosis was confirmed through indirect immunofluorescence by the presence of anti-NMDAR neuronal antibodies in cerebrospinal fluid, the only specific diagnostic test for this condition.^4^

Nearly half of autoimmune psychosis patients may have a pre-existing psychiatric disorder, most commonly depression.^2^ The most common psychiatric symptoms of anti-NMDAR encephalitis are agitation (59%), psychosis (54%), catatonia (42% in adults and 35% in children) and mood disorders (27%).^2^ In 4% of cases, psychiatric symptoms may be the sole presentation.^3^ It is estimated that 37% of cases start before 18 years of age, with 70% showing prodromal flu-like symptoms in the two weeks before onset of encephalitis manifestations.^3^,^4^,^5^ Movement disorders occur in 75% of adults and 95% of children.^3^ As shown in this case report, a lack of typical findings in complementary exams does not rule out diagnosis, although up to 80% of cases show cerebrospinal fluid changes (moderate lymphocytic pleocytosis) and more than 90% show electroencephalogram changes.^2^,^4^

However, cranial magnetic resonance imaging can be normal in up to 50-70% of cases or reveal non-specific hyperintense lesions in up to 50% of later-stage cases and 35% of early-stage cases.^2^,^4^ Electroconvulsive therapy is recognized as an effective and safe first-line treatment for malignant catatonia, leading to complete (60%) or partial (33%) neurological recovery in these patients.^4^,^6^ It also enhances the effectiveness of treatment with immunomodulators, allowing complete remission of psychiatric symptoms and associated risks.^4^,^6^

## Disclosure

The authors report no conflicts of interest.

## Figures and Tables

**Box 1:** List of additional tests conducted in each case

Additional tests	Case 1	Case 2
Laboratory	Toxicological urine tests for cannabis and cocaine and rapid antigen test for COVID-19 (negatives). Biochemical tests: complete blood count, hepatic function tests, renal function, glucose, vitamin D and B12, thyroid function, beta-HCG, serum levels of carbamazepine and lithium, electrolytes, urine test, lipid panel. Serological tests: anti-HIV, HBsAg, Anti-HCV, VDRL (non-reactive). Peripheral autoimmune screening: CRP, ERS, Anti-TPO, TgAb, TRAb, ANA. Neoplastic screening: AFP, LDH, total HCG, CA 125.	Biochemical tests: complete blood count, hepatic function tests, renal function, thyroid function, serum levels of valproate. Peripheral autoimmune screening: CRP, ERS, RF, ANA. Neoplastic screening: AFP, LDH, Beta-hCG.
Cerebrospinal fluid	Opening pressure 145 mmH2O, normal differential cytology (clear and colorless appearance, 3 leukocytes, 0 erythrocytes), chloride 110, glucose 73, total proteins 19.4 mg/dL, fungal testing (negative), VDRL (negative). Indirect Immunofluorescence: anti-NMDA Receptor Antibody: positive for NR1 and NR2.	Leukocytes 30 (Lymphocytes 93%; Monocytes 7%), proteins 64, lactate 13.53, glucose 56, VDRL (negative), oligoclonal bands with exclusive synthesis in cerebrospinal fluid (type II pattern), PCR for herpes zoster (negative).
ECG and EEG	ECG: no abnormalities. Resting EEG: no abnormalities. Video EEG (4 hours): rare epileptiform spikes and waves in the occipital and posterior temporal regions, bilaterally, exclusively during sleep, although the delta-brush pattern was not observed.	EEG (2-3 months of symptoms): no abnormalities. EEG (4-5 months of symptoms): marked disorganization and slowing of baseline activity, with bilateral absence of alpha rhythm. Irregular diffuse theta rhythms of medium amplitude predominated, with a greater projection over the parasagittal areas of both hemispheres, and persistent prominent bifrontal delta waves, without asymmetries. No epileptiform discharges were recorded. Severe diffuse cerebral dysfunction without associated irritative signs.
Imaging tests	Contrast-enhanced head MRI: the brain diffusion study showed no signal abnormalities. Pelvic MRI: no abnormalities. Head/full abdomen/chest CT: no abnormalities. Full abdominal ultrasound: no evidence of abnormalities in the liver, gallbladder, pancreas, spleen, kidneys, abdominal aorta, uterus, ovaries, or bladder. Chest X-ray: interstitial infiltrate and possible subtle consolidations in the lower left lobe.	Head MRI: no abnormalities. Chest CT: no abnormalities. Full abdominal ultrasound: no abnormalities.

AFP = alpha-fetoprotein; ANA = antinuclear antibody; anti-HCV = hepatitis C antibody test; anti-NMDAR = anti-N-methyl-D-aspartate receptor; HCG = human chorionic gonadotropin; CA 125 = cancer antigen 125; CRP = C-reactive protein; CT = computed tomography; ECG = electrocardiogram; EEG = electroencephalogram; ESR = erythrocyte sedimentation rate; HbsAg = hepatitis B surface antigen; LDH = lactate dehydrogenase; MRI = magnetic resonance imaging; PCR = polymerase chain reaction; RF = rheumatoid factor; TgAb = thyroglobulin antibodies; TPO = thyroid peroxidase; TRAb = thyrotropin receptor antibody; VDRL = venereal disease research laboratory.
